# Bis(2-hy­droxy­phen­yl)methanone

**DOI:** 10.1107/S1600536811025438

**Published:** 2011-07-02

**Authors:** Richard Betz, Thomas Gerber, Henk Schalekamp

**Affiliations:** a Nelson Mandela Metropolitan University, Summerstrand Campus, Department of Chemistry, University Way, Summerstrand, PO Box 77000, Port Elizabeth 6031, South Africa

## Abstract

In the title compound, C_13_H_10_O_3_, a benzophenone derivative, the least-squares planes defined by the C atoms of the 2-hy­droxy­phenyl rings inter­sect at an angle of 45.49 (3)°. The substituents on the aromatic systems are both orientated towards the central O atom. Intra- as well as inter­molecular O—H⋯O hydrogen bonds are observed, the latter giving rise to the formation of centrosymmetric dimers. The closest centroid–centroid distance between two π-systems is 3.7934 (7) Å.

## Related literature

For the crystal structure of benzophenone, see: Lobanova (1968 ▶); Kutzke *et al.* (2000 ▶); Fleischer *et al.* (1968 ▶); Bernstein *et al.* (2002 ▶); Moncol & Coppens (2004 ▶). For graph-set analysis of hydrogen bonds, see: Etter *et al.* (1990 ▶); Bernstein *et al.* (1995 ▶). Chelate ligands have found widespread use in coordination chemistry due to the enhanced thermodynamic stability of the resultant coordination compounds in relation to those exclusively applying comparable monodentate ligands, see: Gade (1998 ▶).
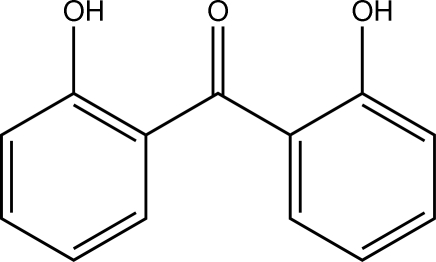


## Experimental

### 

#### Crystal data

C_13_H_10_O_3_
*M* *_r_* = 214.21
Monoclinic, 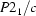

*a* = 7.7371 (2) Å
*b* = 12.2169 (4) Å
*c* = 11.3419 (3) Å
β = 110.610 (2)°
*V* = 1003.46 (5) Å^3^
*Z* = 4
Mo *K*α radiation
μ = 0.10 mm^−1^
*T* = 200 K
0.24 × 0.20 × 0.18 mm

#### Data collection

Bruker APEXII CCD diffractometer
9306 measured reflections
2483 independent reflections
1939 reflections with *I* > 2σ(*I*)
*R*_int_ = 0.033

#### Refinement

*R*[*F* ^2^ > 2σ(*F* ^2^)] = 0.038
*wR*(*F* ^2^) = 0.106
*S* = 1.05
2483 reflections
147 parameters
H-atom parameters constrained
Δρ_max_ = 0.27 e Å^−3^
Δρ_min_ = −0.19 e Å^−3^

### 

Data collection: *APEX2* (Bruker, 2010 ▶); cell refinement: *SAINT* (Bruker, 2010 ▶); data reduction: *SAINT*; program(s) used to solve structure: *SHELXS97* (Sheldrick, 2008 ▶); program(s) used to refine structure: *SHELXL97* (Sheldrick, 2008 ▶); molecular graphics: *ORTEP-3* (Farrugia, 1997 ▶) and *Mercury* (Macrae *et al.*, 2008 ▶); software used to prepare material for publication: *SHELXL97* and *PLATON* (Spek, 2009 ▶).

## Supplementary Material

Crystal structure: contains datablock(s) I, global. DOI: 10.1107/S1600536811025438/im2302sup1.cif

Supplementary material file. DOI: 10.1107/S1600536811025438/im2302Isup2.cdx

Structure factors: contains datablock(s) I. DOI: 10.1107/S1600536811025438/im2302Isup3.hkl

Supplementary material file. DOI: 10.1107/S1600536811025438/im2302Isup4.cml

Additional supplementary materials:  crystallographic information; 3D view; checkCIF report

## Figures and Tables

**Table 1:** Hydrogen-bond geometry (Å, °)

*D*—H⋯*A*	*D*—H	H⋯*A*	*D*⋯*A*	*D*—H⋯*A*
O2—H2⋯O1	0.84	1.88	2.6061 (11)	144
O2—H2⋯O1^i^	0.84	2.44	2.9976 (12)	124
O3—H3⋯O1	0.84	1.95	2.6623 (11)	142

Symmetry code: (i) 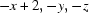.

**Fig. 1.**
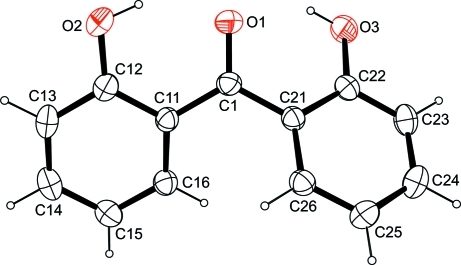
The molecular structure of the title compound, with atom labels and anisotropic displacement ellipsoids (drawn at 50% probability level).

**Fig. 2.**
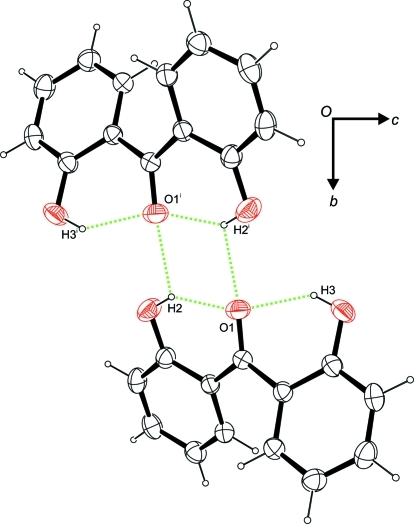
Intermolecular contacts, viewed along [-1 0 0]. Symmetry operator: i -x + 2, -y, -z.

**Fig. 3.**
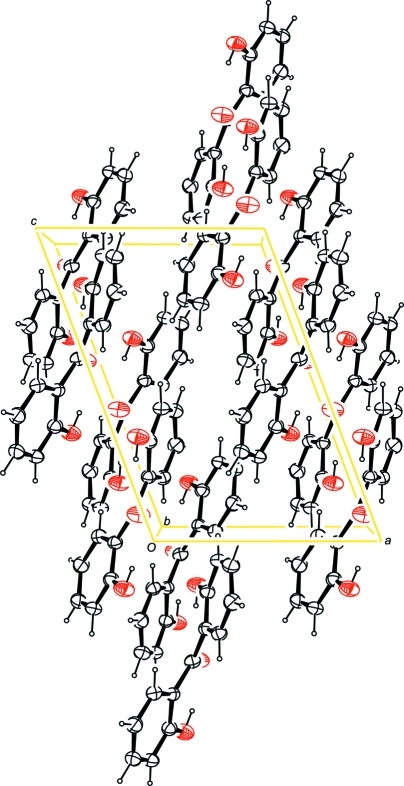
Molecular packing of the title compound, viewed along [0 1 0] (anisotropic displacement ellipsoids drawn at 50% probability level).

## supplementary crystallographic information

### Comment

Chelate ligands have found widespread use in coordination chemistry due to the enhanced thermodynamic stability of resultant coordination compounds in relation to coordination compounds exclusively applying comparable monodentate ligands (Gade, 1998). Combining two identical donor atoms in different states of hybridization seemed to be useful to us to accomodate a large variety of metal centers of variable Lewis acidity. To enable comparative studies in terms of bond lengths and angles in envisioned coordination compounds, we determined the molecular and crystal structure of the title compound. The crystal structure of benzophenone is apparent in the literature (Lobanova, 1968; Kutzke *et al.*, 2000; Fleischer *et al.*, 1968; Bernstein *et al.*, 2002; Moncol & Coppens, 2004).

The title compound is a symmetrical substitution product of benzophenone bearing one hydroxyl group in *ortho*-position of each phenyl ring. Both aromatic moieties adopt a conformation in which the substituents are orientated towards the central oxygen atom. The least-squares planes defined by the respective carbon atoms of both *ortho*-hydroxyphenyl rings intersect at an angle of 45.49 (3) °. Intracyclic C–C–C angles hardly deviate from the ideal value of 120 °.

In the crystal structure, intra- as well as intermolecular hydrogen bonds are observed. In both cases, the *sp*^2^-hybridized oxygen atom acts as acceptor, but while one of the hydroxyl groups exclusively forms an intramolecular hydrogen bond, the other hydroxyl group forms a bifurcated hydrogen bond to the keto group's oxygen atom of a neighbouring molecule as well. In total, two molecules are connected to centrosymmetric dimers. The descriptor for the hydrogen bonding system in terms of graph-set analysis (Etter *et al.*, 1990; Bernstein *et al.*, 1995) is *DDR*^2^_2_(12) on the unitary level. The shortest intercentroid distance between two π-systems is 3.7934 (7) Å and is apparent between two different aromatic moieties.

The packing of the title compound in the crystal structure is shown in Figure 3.

### Experimental

The compound was obtained commercially (Aldrich). Crystals suitable for the X-ray diffraction study were taken directly from the provided product.

### Refinement

Carbon-bound H atoms were placed in calculated positions (C—H 0.95 Å) and were included in the refinement in the riding model approximation, with *U*(H) set to 1.2*U*_eq_(C). The hydrogen atoms of the hydroxyl groups were allowed to rotate with a fixed angle around the O–C bonds to best fit the experimental electron density (HFIX 147 in the *SHELX* program suite (Sheldrick, 2008).

### Crystal data

**Table:**

C_13_H_10_O_3_	*F*(000) = 448
*M**_r_* = 214.21	*D*_x_ = 1.418 Mg m^−^^3^
Monoclinic, *P*2_1_/*c*	Mo *K*α radiation, λ = 0.71073 Å
Hall symbol: -P 2ybc	Cell parameters from 4455 reflections
*a* = 7.7371 (2) Å	θ = 2.5–28.3°
*b* = 12.2169 (4) Å	µ = 0.10 mm^−^^1^
*c* = 11.3419 (3) Å	*T* = 200 K
β = 110.610 (2)°	Platelet, colourless
*V* = 1003.46 (5) Å^3^	0.24 × 0.20 × 0.18 mm
*Z* = 4	

### Data collection

**Table:**

Bruker APEXII CCD diffractometer	1939 reflections with *I* > 2σ(*I*)
Radiation source: fine-focus sealed tube	*R*_int_ = 0.033
graphite	θ_max_ = 28.3°, θ_min_ = 2.5°
φ and ω scans	*h* = −9→10
9306 measured reflections	*k* = −15→16
2483 independent reflections	*l* = −15→15

### Refinement

**Table:**

Refinement on *F*^2^	Primary atom site location: structure-invariant direct methods
Least-squares matrix: full	Secondary atom site location: difference Fourier map
*R*[*F*^2^ > 2σ(*F*^2^)] = 0.038	Hydrogen site location: inferred from neighbouring sites
*wR*(*F*^2^) = 0.106	H-atom parameters constrained
*S* = 1.05	*w* = 1/[σ^2^(*F*_o_^2^) + (0.0563*P*)^2^ + 0.1145*P*] where *P* = (*F*_o_^2^ + 2*F*_c_^2^)/3
2483 reflections	(Δ/σ)_max_ < 0.001
147 parameters	Δρ_max_ = 0.27 e Å^−^^3^
0 restraints	Δρ_min_ = −0.19 e Å^−^^3^

### Fractional atomic coordinates and isotropic or equivalent isotropic displacement parameters (Å^2^)

**Table:**

	*x*	*y*	*z*	*U*_iso_*/*U*_eq_	
O1	0.97092 (13)	0.09617 (6)	0.08169 (7)	0.0405 (2)	
O2	0.78137 (13)	0.09971 (7)	−0.15911 (8)	0.0407 (2)	
H2	0.8528	0.0719	−0.0919	0.061*	
O3	1.09865 (13)	0.09673 (6)	0.33247 (8)	0.0407 (2)	
H3	1.0534	0.0664	0.2617	0.061*	
C1	0.94988 (15)	0.19703 (8)	0.08687 (9)	0.0271 (2)	
C11	0.80678 (14)	0.25299 (8)	−0.01695 (9)	0.0255 (2)	
C12	0.73030 (15)	0.20048 (9)	−0.13533 (10)	0.0294 (2)	
C13	0.59669 (16)	0.25366 (10)	−0.23411 (10)	0.0348 (3)	
H13	0.5499	0.2199	−0.3146	0.042*	
C14	0.53150 (16)	0.35473 (10)	−0.21639 (11)	0.0358 (3)	
H14	0.4388	0.3896	−0.2844	0.043*	
C15	0.60019 (16)	0.40645 (9)	−0.09954 (10)	0.0334 (3)	
H15	0.5537	0.4759	−0.0876	0.040*	
C16	0.73588 (15)	0.35604 (9)	−0.00167 (10)	0.0287 (2)	
H16	0.7827	0.3915	0.0779	0.034*	
C21	1.06958 (14)	0.25691 (8)	0.19946 (9)	0.0253 (2)	
C22	1.13591 (15)	0.20282 (9)	0.31684 (9)	0.0283 (2)	
C23	1.24280 (15)	0.25933 (10)	0.42392 (9)	0.0340 (3)	
H23	1.2807	0.2242	0.5037	0.041*	
C24	1.29406 (17)	0.36599 (10)	0.41490 (11)	0.0372 (3)	
H24	1.3674	0.4039	0.4887	0.045*	
C25	1.23981 (16)	0.41868 (9)	0.29931 (11)	0.0341 (3)	
H25	1.2798	0.4913	0.2933	0.041*	
C26	1.12737 (15)	0.36495 (8)	0.19308 (10)	0.0282 (2)	
H26	1.0883	0.4017	0.1142	0.034*	

### Atomic displacement parameters (Å^2^)

**Table:**

	*U*^11^	*U*^22^	*U*^33^	*U*^12^	*U*^13^	*U*^23^
O1	0.0588 (6)	0.0236 (4)	0.0317 (4)	0.0033 (4)	0.0067 (4)	−0.0005 (3)
O2	0.0478 (5)	0.0359 (5)	0.0319 (4)	0.0011 (4)	0.0058 (4)	−0.0106 (3)
O3	0.0538 (6)	0.0312 (4)	0.0305 (4)	−0.0037 (4)	0.0068 (4)	0.0094 (3)
C1	0.0338 (6)	0.0232 (5)	0.0247 (5)	−0.0006 (4)	0.0107 (4)	0.0006 (4)
C11	0.0279 (5)	0.0258 (5)	0.0223 (5)	−0.0033 (4)	0.0081 (4)	0.0005 (4)
C12	0.0301 (5)	0.0315 (5)	0.0266 (5)	−0.0041 (4)	0.0101 (4)	−0.0028 (4)
C13	0.0319 (6)	0.0471 (7)	0.0217 (5)	−0.0048 (5)	0.0051 (4)	−0.0022 (5)
C14	0.0293 (6)	0.0478 (7)	0.0282 (5)	0.0025 (5)	0.0073 (5)	0.0099 (5)
C15	0.0340 (6)	0.0331 (6)	0.0341 (6)	0.0052 (5)	0.0131 (5)	0.0051 (5)
C16	0.0312 (5)	0.0296 (5)	0.0254 (5)	−0.0006 (4)	0.0099 (4)	0.0004 (4)
C21	0.0268 (5)	0.0258 (5)	0.0230 (5)	0.0023 (4)	0.0083 (4)	0.0012 (4)
C22	0.0290 (5)	0.0293 (5)	0.0264 (5)	0.0024 (4)	0.0097 (4)	0.0040 (4)
C23	0.0333 (6)	0.0448 (7)	0.0218 (5)	0.0014 (5)	0.0074 (5)	0.0038 (5)
C24	0.0355 (6)	0.0454 (7)	0.0279 (6)	−0.0065 (5)	0.0078 (5)	−0.0082 (5)
C25	0.0354 (6)	0.0313 (6)	0.0356 (6)	−0.0059 (5)	0.0125 (5)	−0.0042 (5)
C26	0.0304 (5)	0.0275 (5)	0.0269 (5)	0.0012 (4)	0.0101 (4)	0.0019 (4)

### Geometric parameters (Å, °)

**Table:**

O1—C1	1.2470 (12)	C15—C16	1.3760 (15)
O2—C12	1.3489 (13)	C15—H15	0.9500
O2—H2	0.8400	C16—H16	0.9500
O3—C22	1.3530 (13)	C21—C26	1.4035 (14)
O3—H3	0.8400	C21—C22	1.4112 (13)
C1—C11	1.4703 (14)	C22—C23	1.3886 (15)
C1—C21	1.4802 (14)	C23—C24	1.3763 (16)
C11—C16	1.4081 (15)	C23—H23	0.9500
C11—C12	1.4161 (14)	C24—C25	1.3864 (16)
C12—C13	1.3894 (15)	C24—H24	0.9500
C13—C14	1.3750 (17)	C25—C26	1.3790 (15)
C13—H13	0.9500	C25—H25	0.9500
C14—C15	1.3936 (16)	C26—H26	0.9500
C14—H14	0.9500		
			
C12—O2—H2	109.5	C15—C16—H16	119.3
C22—O3—H3	109.5	C11—C16—H16	119.3
O1—C1—C11	119.72 (9)	C26—C21—C22	118.19 (9)
O1—C1—C21	118.46 (9)	C26—C21—C1	122.29 (9)
C11—C1—C21	121.81 (9)	C22—C21—C1	119.46 (9)
C16—C11—C12	118.09 (9)	O3—C22—C23	116.77 (9)
C16—C11—C1	122.23 (9)	O3—C22—C21	123.28 (9)
C12—C11—C1	119.62 (9)	C23—C22—C21	119.94 (10)
O2—C12—C13	116.92 (9)	C24—C23—C22	120.28 (10)
O2—C12—C11	123.30 (10)	C24—C23—H23	119.9
C13—C12—C11	119.78 (10)	C22—C23—H23	119.9
C14—C13—C12	120.60 (10)	C23—C24—C25	120.71 (10)
C14—C13—H13	119.7	C23—C24—H24	119.6
C12—C13—H13	119.7	C25—C24—H24	119.6
C13—C14—C15	120.62 (10)	C26—C25—C24	119.54 (10)
C13—C14—H14	119.7	C26—C25—H25	120.2
C15—C14—H14	119.7	C24—C25—H25	120.2
C16—C15—C14	119.44 (11)	C25—C26—C21	121.14 (10)
C16—C15—H15	120.3	C25—C26—H26	119.4
C14—C15—H15	120.3	C21—C26—H26	119.4
C15—C16—C11	121.37 (10)		
			
O1—C1—C11—C16	159.44 (10)	O1—C1—C21—C26	146.95 (11)
C21—C1—C11—C16	−19.72 (15)	C11—C1—C21—C26	−33.88 (15)
O1—C1—C11—C12	−17.66 (15)	O1—C1—C21—C22	−30.02 (14)
C21—C1—C11—C12	163.18 (10)	C11—C1—C21—C22	149.15 (10)
C16—C11—C12—O2	−176.94 (9)	C26—C21—C22—O3	−175.80 (10)
C1—C11—C12—O2	0.28 (16)	C1—C21—C22—O3	1.29 (16)
C16—C11—C12—C13	3.58 (15)	C26—C21—C22—C23	5.22 (15)
C1—C11—C12—C13	−179.20 (9)	C1—C21—C22—C23	−177.68 (9)
O2—C12—C13—C14	177.36 (10)	O3—C22—C23—C24	176.91 (10)
C11—C12—C13—C14	−3.13 (17)	C21—C22—C23—C24	−4.05 (17)
C12—C13—C14—C15	0.97 (17)	C22—C23—C24—C25	0.12 (18)
C13—C14—C15—C16	0.68 (17)	C23—C24—C25—C26	2.53 (18)
C14—C15—C16—C11	−0.13 (17)	C24—C25—C26—C21	−1.22 (17)
C12—C11—C16—C15	−1.98 (15)	C22—C21—C26—C25	−2.61 (16)
C1—C11—C16—C15	−179.12 (10)	C1—C21—C26—C25	−179.62 (10)

### Hydrogen-bond geometry (Å, °)

**Table:**

*D*—H···*A*	*D*—H	H···*A*	*D*···*A*	*D*—H···*A*
O2—H2···O1	0.84	1.88	2.6061 (11)	144
O2—H2···O1^i^	0.84	2.44	2.9976 (12)	124
O3—H3···O1	0.84	1.95	2.6623 (11)	142

Symmetry codes: (i) −*x*+2, −*y*, −*z*.
